# Don’t Wet a Lead Pencil

**Published:** 1898-02

**Authors:** 


### Don't Wet a Lead Pencil.

The practice of wetting a lead pencil on the tongue before using it is an unclean habit, to say the least, and perhaps also a dangerous one.

Recently a woman of fine bearing and elegantly dressed stepped into the counting-room of one of the local papers of a large city to insert an advertisement. Having no pencil of her own, she picked up a pencil which was tied with a string to a pad used for writing. At once she moistened the lead with her tongue and began to write.

An elderly woman who was standing by reminded her that the pencil had just been used by an old man, ragged and dirty, greasy and filthy, who also had contracted the same habit of wetting the pencil on his tongue every time he wrote a word. The disgusted woman flung the pencil away and scolded the young man behind the counter until he sharpened a brand new pencil for her use and benefit.

The habit is a foolish one. Instead of making the pencil write more freely and easily, it hardens it and makes it write blurred and irregular.

Newspaper men and those who use lead pencils a great deal never dampen the lead in the mouth or with a sponge. Besides being injurious to the lead it is a dangerous habit, inasmuch as disease has been known to be conveyed in that way into the system.—*Indian Lancet.*

